# Antibodies targeting the European lobster (*Palinurus elephas*) vitellogenin developed by mRNA isolation and in-silico-designed antigenic peptides

**DOI:** 10.1242/bio.059019

**Published:** 2022-05-13

**Authors:** Faustina B. Cannea, Maria Cristina Follesa, Cristina Porcu, Rossano Rossino, Alessandra Olianas, Antonio Rescigno, Alessandra Padiglia

**Affiliations:** 1 Dipartimento di Scienze della Vita e dell'Ambiente (DiSVA), Biomedical section, University of Cagliari, Cittadella Universitaria di Monserrato, 09042 Monserrato, Cagliari, Italy; 2 Dipartimento di Scienze della Vita e dell'Ambiente (DiSVA), Marine Bioecology section, University of Cagliari, via T. Fiorelli 1, 09126 Cagliari, Italy; 3 Dipartimento di Scienze Mediche e Sanità Pubblica (DSMSP), University of Cagliari, AOU Presidio microcitemico via Jenner, 09121 Cagliari, Italy; 4 Dipartimento di Scienze Biomediche (DiSB), Cittadella Universitaria di Monserrato, 09042 Monserrato, Cagliari, Italy

**Keywords:** *Palinurus elephas*, Vitellogenin, Antibody anti-vitellogenin, ELISA, mRNA, CODEHOP

## Abstract

Vitellogenin is an essential protein involved in ovary maturation in many animals. Detection of this protein correlated with reproductive capacity may be important if carried out on marine organisms such as the red spiny lobster *Palinurus elephas*, a crustacean that is an economically important crop from wild fish catches. Moreover, in recent years, vitellogenin has assumed an important role as a possible biomarker of marine environmental pollution, as its expression levels can be influenced by the presence of similar estrogen pollutants and can affect the reproductive sphere of marine organisms such as crustaceans. The *P. elephas* vitellogenin protein and its coding gene have never been isolated, so there is little information about its presence in this lobster. The aim of the present study was to develop a molecular strategy to create, for the first time, an antibody for the detection and quantization of vitellogenin in *P. elephas*.

## Abstract (precis)

**Summary:** The development of anti-vitellogenin antibodies of *Palinurus elephas* could be applied to building a non-invasive reading system for monitoring the reproductive status of these crustaceans and as a useful tool for controlling the quality of their aquatic environment.

## INTRODUCTION

The determination of vitellogenin (VTG) levels in marine aquatic species has for some time aroused much interest from both fishery and environmental points of view. The study of VTG has now definitively found wide application in controlling the reproductive biology of fish, including the management of natural populations, the development of appropriate breeding practices and quality control of the aquatic environment (Hiramatsu et al., 2002). VTG is a physiological indicator of the maturation stage of the female gonad (Reading et al., 2017), therefore, the possibility of measuring its concentration variations in many species throughout the life cycle makes VTG a far more sensitive indicator of sexual development with respect to endocrine parameters such as oestradiol, testosterone and gonadotropins (Denslow et al., 1999; Matozzo et al., 2008). VTG is a glycolipophosphoprotein (300–700 kDa) synthesized in the reproductive phase of many vertebrate and invertebrate animals. Expressed in the ovary and in the extraovarian sites of females in decapod crustaceans (Guan et al., 2016; Jia et al., 2013), VTG exhibits similar molecular features in phylogenetically related organisms, signifying its important physiological role in the course of evolution (Chen et al., 1997). Extraovarian synthesis involves the transport of VTG into the ovary through a blood vessel or hemolymph, in which it is internalized in the oocyte via receptor-mediated endocytosis (Barber et al., 1991; Ruan et al., 2020). Inside the oocyte, VTG undergoes a post-translational modification that consists of glycosylation events, the addition of lipids and proteolytic cleavages that generate smaller proteins collectively called vitelline (Vt), which are the main protein components for the development and maturation of eggs (Meusy and Payen, 1988; Wilder et al., 2002; Tiu et al., 2008). The proteolytic cuts originating from Vt occur in specific domains of the VTG precursor. The VTG amino acid sequence of the lobster *Homarus americanus* (HaVg1) deduced from cDNA highlighted the presence of several potential cleavage sites of the subtilisin endopeptidase (characterized by the consensus sequences RXXR or RXK/RK/R) from which at least three subunits with estimated molecular sizes of 80, 105 and 90 kDa could be produced starting from the precursor (Tiu et al., 2009). The precursor VTG is expressed at low levels in males and immature females, while it reaches high levels of expression during oogenesis in mature females (Thongda et al., 2015). Estrogen 17 β-oestradiol and estrogen-like molecules are powerful inducers of vitellogenesis in immature females since they can stimulate the expression of VTG (Palmer et al., 1998). In males, the genes for VTG (Vtgs) are normally silent, but in response to estrogen or estrogen-like substances, they undergo excessive transcriptional activity, and as a result, the protein synthesis occurs in the liver or functionally similar organs. Abnormal expression of this protein in males makes VTG a strong biomarker due to its ability to undergo variations in gene expression in aquatic species that live in aquatic environments contaminated by estrogens and estrogen-like molecules (Denslow et al., 1999; Sumpter and Jobling, 1995). In recent decades, greater attention has been given to assessing the adverse effects of chemicals that interfere with the endocrine system (EDCs: endocrine disrupting chemicals) in aquatic environments (Park et al., 2019). The concentration of VTG measured in the plasma of male fish under physiological conditions is 10–50 ng/ml, while in reproductive females, it is approximately 20 mg/ml (Denslow et al., 1999; Folmar et al., 1996; Parks et al., 1999). Studies on *H. americanus* have shown that VTG was undetectable in hemolymph from adult males, however, it increased 40-fold during the reproductive phase (Tsukimura et al., 2002). Since VTG levels have been shown to be closely related to the reproductive conditions of different crustaceans (Tsukimura, 2001; Tsukimura et al., 2002), their quantification could be a useful tool for monitoring the reproductive status of species living in specific environments. The European lobster *Palinurus elephas* (Fabricius, 1787) is a long-lived and slow growing species typical of temperate waters and is widely distributed in the Mediterranean Sea and the Atlantic Ocean. The development of increasingly powerful and efficient fishing methods in recent years has led to a decrement in the stocks of this crustacea in Sardinia (Italy) and other countries of the Mediterranean basin (Cau et al., 2019; Follesa et al., 2009).

The main objective of this research was to develop a specific antibody for the detection of VTG in the eggs of the *P. elephas* lobster since the study of this protein has now definitively found wide application not only in controlling the reproductive biology of marine animals but also in monitoring the quality of aquatic environments. A specific antibody for *P. elephas* VTG could be a useful tool for the following reasons: (1) to investigate the reproductive suitability of the population of this crustacean since VTG is a good indicator of female reproductive activity, and (2) to evaluate non-physiological protein amounts in males since VTG is considered a biomarker of environment pollution. Moreover, the isolation of mRNA coding for VTG allowed us, for the first time in this crustacean, to enrich the gene databases with a part of the coding sequence from which it was possible to derive the corresponding primary structure of the encoded protein.

## RESULTS

**Fig. 1.**
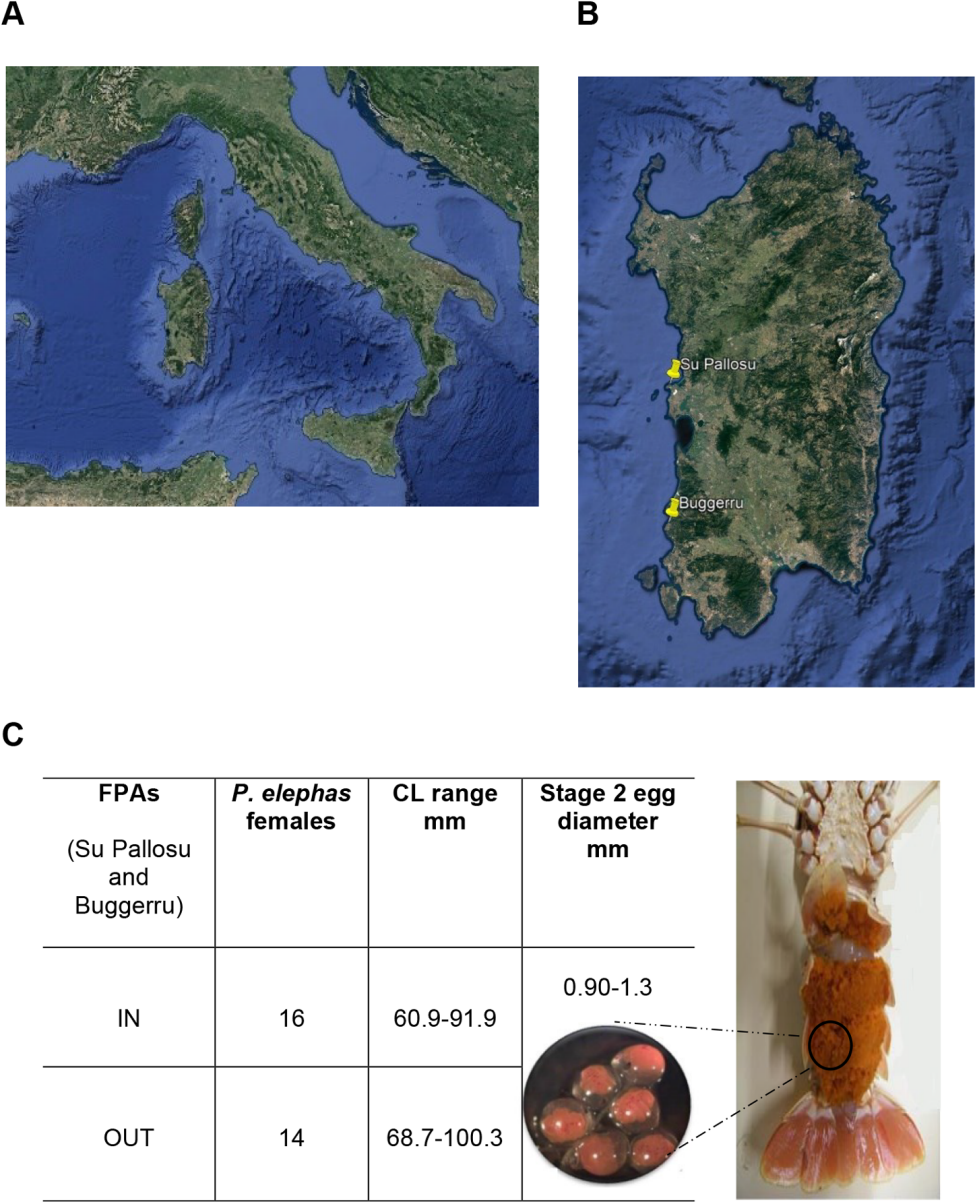
**(A) Satellite view, obtained through Google Earth, of the Mediterranean Sea, where Sardinia occupies a central position.** (B) Sardinia island with the position of FPAs Su Pallosu (central western coast) and Buggerru (southwestern coast) shown with yellow points. (C) Number of ovigerous *P. elephas* females (eggs stage 2) captured inside (IN) and outside (OUT) FPAs, as well as their size range and egg diameter. The presence of external eggs on the pleopods and carapace length (CL) were used as indicators of maturity.

In this study, Mediterranean *P. elephas* females with external eggs in the intermediate stage (stage 2) were selected since the levels of VTG in crustaceans appear to decrease significantly with the beginning of the oviposition of the animals (stage 3) (Tsukimura et al., 2002). The animals were captured inside and outside of two fully protected areas (FPAs) located on the western coast of Sardinia island (Italy) in the middle of the Mediterranean Sea (Fig. 1A). Specifically, the FPAs considered were Su Pallosu located on the central western coast of Sardinia and Buggerru on its southwestern coast (Fig. 1A,B). The mRNA was obtained and VTG concentration was estimated from the egg clutches of 30 ovigerous females at the same stage of intermediate development (stage 2) as follows: 16 ovigerous females were caught inside of FPAs and 14 outside of FPAs, all at the same developmental stage (Fig. 1C). The size of external eggs on the pleopods (Fig. 1C) and carapace length (CL) was used as an indicator of maturity (Kang et al., 2008).

### *Palinurus elephas* Vtg cDNA amplification and antigenic peptide synthesis

**Fig. 2.**
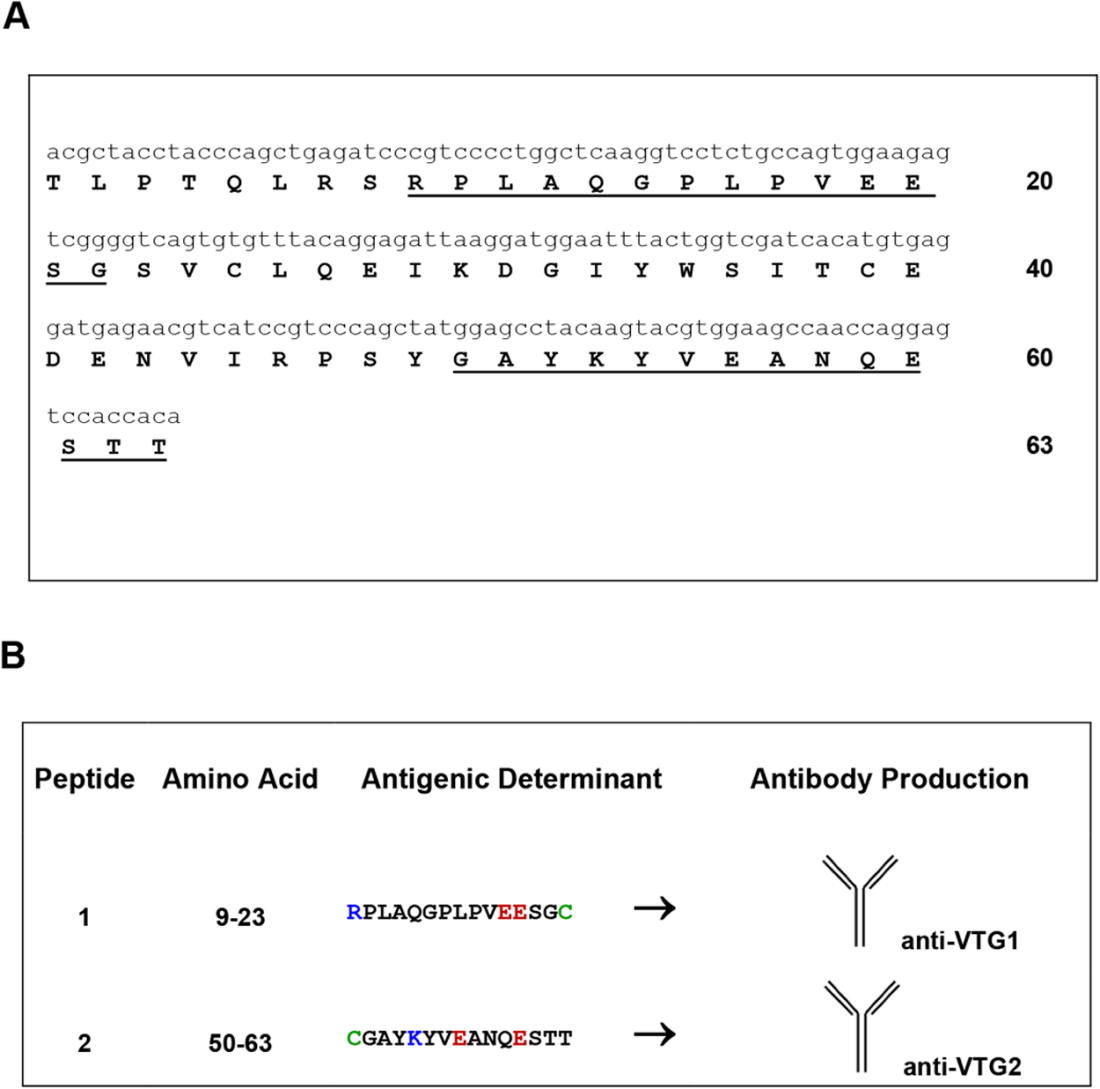
**(A) Nucleotides and, below, deduced amino acid sequences of VTG from *P. elephas*.** The underlined regions are predicted to be antigenic regions. (B) Information related to synthetic peptides. An extra ‘C’ (highlighted in green) is added to the C terminus (or N terminus) to facilitate conjugation. The antigenic positively charged residues (K, R) and negatively charged residues (E) are in blue and in red, respectively. The synthesis and epitope predictions were made using GenScript. Residues of VTG enzyme and synthetic peptides are reported with the one-letter code.

As a first step, mRNA isolated from eggs was reverse transcribed and subsequently subjected to PCR that utilized copies of partially degenerated primers. In particular, the primers employed were designed with the CODEHOP strategy (Rose et al., 2003), starting from an alignment of multiple sequences of VTG proteins (Fig. S1). The results of the PCR fragment sequencing allowed us to reconstruct, in silico, a part of the primary structure of the protein corresponding to a peptide of 63 amino acids (Fig. 2A). By aligning the sequence obtained with protein sequences deposited in NCBI databases (https://blast.ncbi.nlm.nih.gov/Blast.cgi) and UniProt (https://www.UniProt.org/), we found a homology identity of 60–70% with the amino terminal portion of other crustacean VTGs (Fig. S2). An analysis of the amino acid sequence to establish the presence of antigenic regions revealed the potential presence of two epitopes. This allowed the biotechnological synthesis of two peptides, the antigenic peptide 1 AA 9-AA23 and antigenic peptide 2 AA50-AA63 (Fig. 2B), which were used to create two different antibodies (anti-VTG1 and anti-VTG2) for ELISA experiments to detect the VTG expressed in the females of *P. elephas* in the second stage of egg maturation.

### Purity of the anti-VTG1 and anti-VTG2 antibodies

**Fig. 3.**
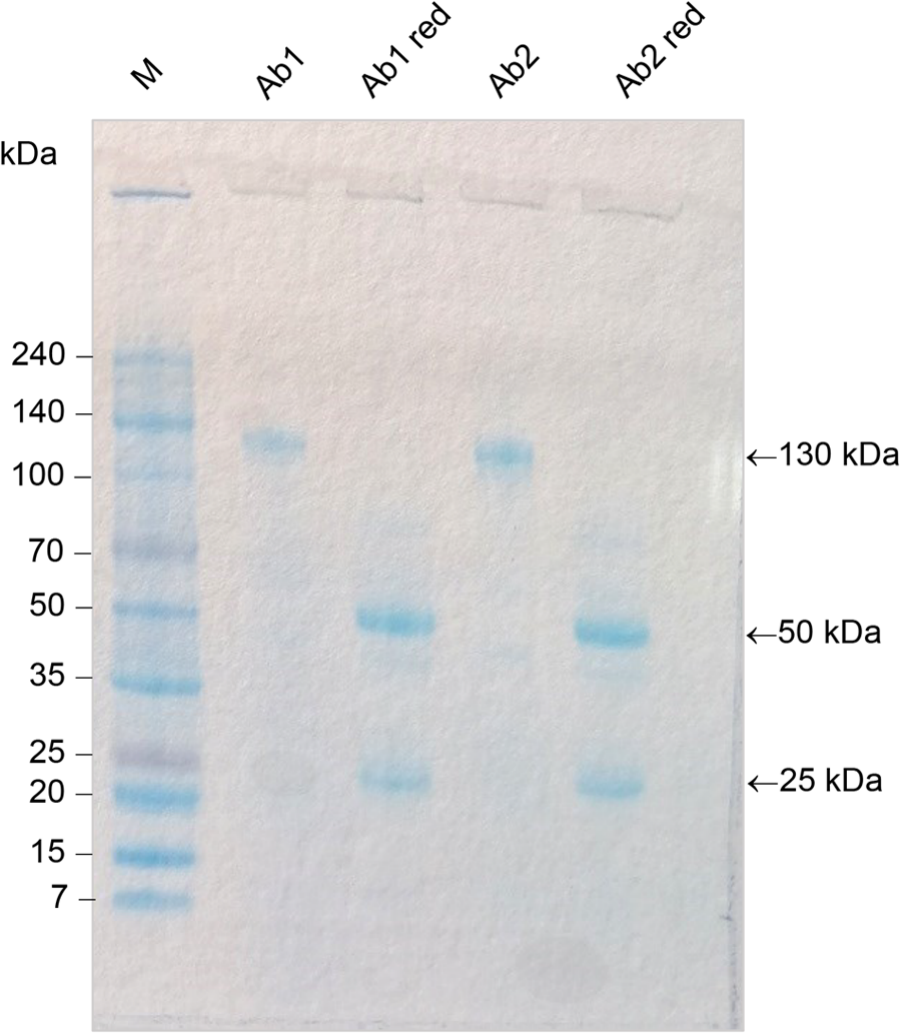
**SDS-PAGE of *P. elephas* anti-VTG1 and anti-VTG-2 antibodies.** The electrophoretic profile obtained under non-reducing conditions indicates the presence of a single band of about 130 kDa for anti-VTG1 (Ab1) and anti-VTG2 (Ab2). Bands of about 25 and 50 kDa (lanes Ab1 red and Ab2 red) were obtained for each antibody after treatment with β-mercaptoethanol. The arrows (←) indicate the anti-bodies anti-VTG bands. The size of the bands (kDa) of the protein ladder (M) is reported on the left.

The purity of the anti-VTG1 and anti-VTG2 antibodies was evaluated through the conducted SDS–PAGE experiments. The electrophoretic profile obtained under nonreducing conditions indicated the presence of a single band of about 130 kDa. As expected, in the presence of reducing conditions after treatment with β-mercaptoethanol, for each antibody, we highlighted two bands of about 25 and 50 kDa, corresponding to the light and heavy chain, respectively (Fig. 3).

### ELISA experiments

**Fig. 4.**
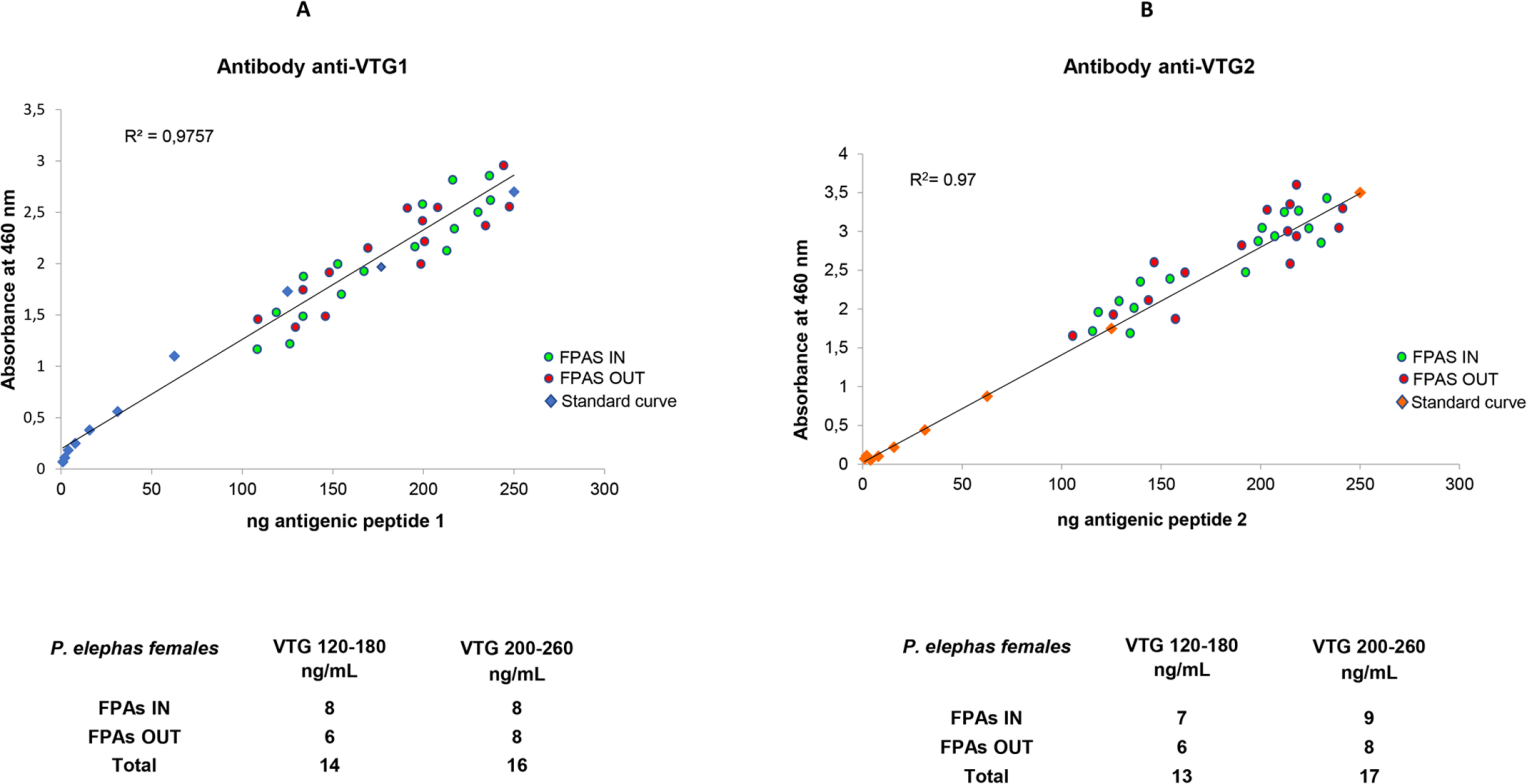
**(A) Dispersion plot illustrating the distribution of VTG concentrations obtained with the ELISA using anti-VTG1and (B) anti VTG2.** The red dots refer to VTG determinations carried out on the samples of eggs of females captured outside the FPAs, while the green dots refer to determinations carried out on the egg fields of females captured inside the FPAs. The standard curve (A) was obtained using antigenic peptide 1 (*R*^2^=0.9757) in the presence of the anti-VTG1 antibody. The standard curve (B) was obtained using antigenic peptide 2 (*R*^2^=0.9757) in the presence of the anti-VTG2 antibody. For both antigenic peptides concentrations used were 250, 125, 62.5, 31.25, 15.65, 7.81, 3.9, 1.95 and 0.97 ng/ml. Samples were read at 460 nm. (B) The number of ovigerous females and the estimated VTG concentration range inside and outside FPAs (A,B). The VTG concentration's relationship with both the inside and outside FPAs did not indicate any statistical differences. Fisher's exact test statistic=1; *P*>0.05.

Different concentrations of peptide 1 or peptide 2 were used in ELISA experiments in the presence of specific antibodies (anti-VTG1 or anti-VTG2) to construct the standard reference curve (Fig. 4A,B). The standard curve R^2^ values obtained were the same for antigenic peptide 1 (R^2^=0.9757) and peptide 2 (R^2^=0.97). The biological samples used in ELISA experiments were obtained from 30 females and six males of *P. elephas* captured inside and outside the FPAs. The external eggs attached to the pleopods were removed from the females, and the hemolymph was taken from the males. By relating the absorbance of each sample with an unknown concentration to the previously established standard curve, we obtained the amounts (ng/ml) of VTG contained in the eggs of *P. elephas*. These were found in a range of 200–260 ng in 14 of the 30 females analyzed, while in the other 16, the concentrations ranged from 120–180 ng/ml (Fig. 4A,B). Differences in VTG levels in stage 2 eggs could be correlated with the sexual maturity of females within the same stage of development. The VTG concentration did not vary between the females captured inside (IN) and outside (OUT) FPAs. In fact, an analysis of the VTG concentration in relationship with the inside and outside FPAs did not show any statistical differences (Fisher's exact test statistic=1; *P*>0.05). When compared to those found for *H. americanus* males (Tsukimura et al., 2002), the absorbance values obtained for *P. elephas* males were so low so as to make it impossible to calculate concentration values to correlate with the standard curve. All ELISA experiments were conducted in parallel using both available antibodies, and the VTG concentrations detected were the same in the presence of each anti-VTG antibody.

### Western blot experiments

**Fig. 5.**
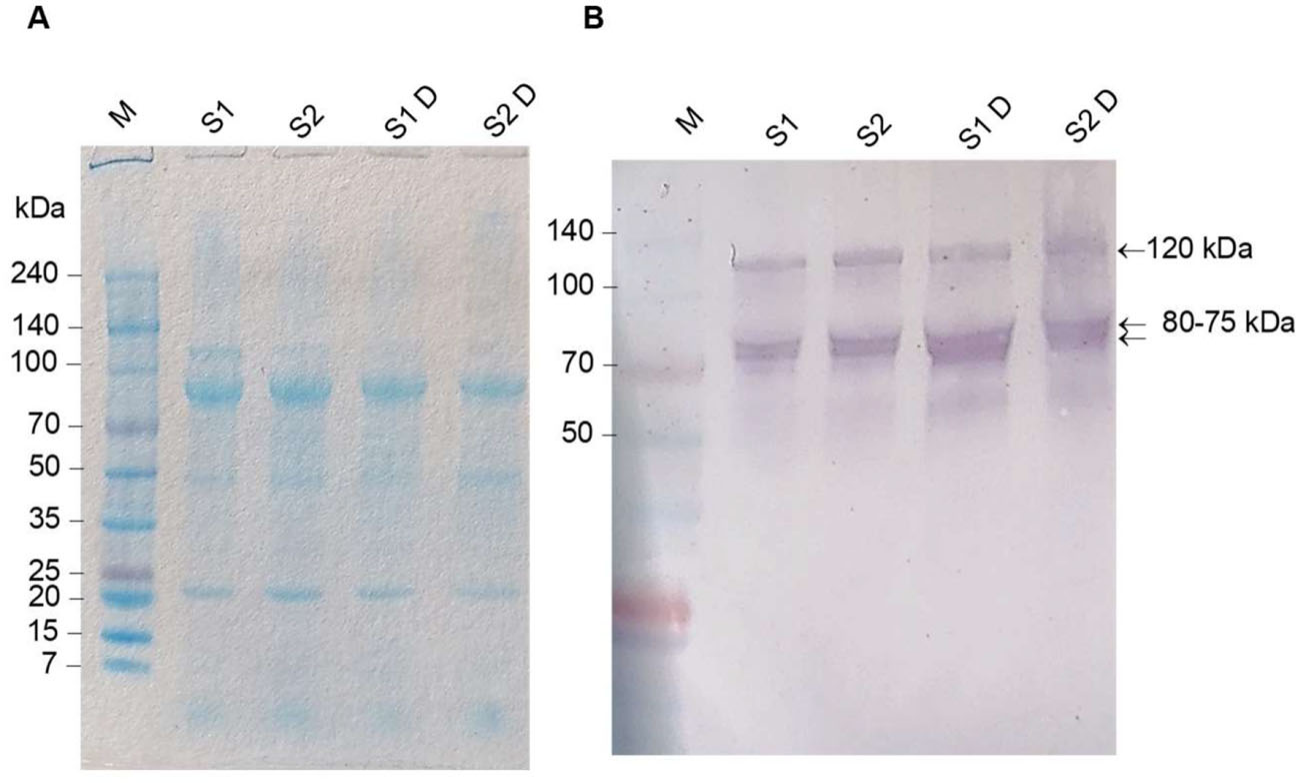
**Results of SDS-PAGE (A) and western blot analysis using anti-VTG1 antibody (B).** An aliquot of all homogenate samples with a concentration of VTG120-180 ng/ml were pooled into one sample (S1). Similarly, an aliquot of all samples with a concentration of VTG 200-260 ng/ml (S2) was pooled. The same S1 and S2 samples were digested with the subtilisin protease (S1D and S2D samples). The SDS-PAGE (A) and western blot profiles (B) did not show any particular differences between the samples treated and untreated with the protease. The different concentrations of VTG detected by ELISA were also not reflected in the number of immunoreactive bands to the anti-VTG1 antibody. The arrows indicate the presence of an immunoreactive band of about 120 kDa and two very close bands between 80–75 kDa. Similar results were obtained using anti-VTG2 antibody (Fig. S3).

The total protein of *P. elephas* egg homogenates separated by SDS–PAGE revealed numerous bands with molecular masses between 20 and 120 kDa (Fig. 5A). To establish which protein band was immunoreactive to the anti-VTG1 and anti-VTG2 antibodies, we conducted western blot experiments. In the presence of anti-VTG1 or anti-VTG2 antibodies, immunoblotting showed the same band profiles, with an immunoreactive protein of about 120 kDa, as well as two very close bands in the 70–80 kDa range. To understand whether any of these proteins could undergo further digestion and release products detectable by anti-VTG1 and anti-VTG2 antibodies, we subjected the homogenate samples to proteolytic digestion with subtilisin. The experiments were repeated numerous times in the presence of proteases and showed a profile of immunoreactive bands that was very similar to that obtained for the samples not subjected to digestion. The results obtained with anti-VTG1 antibody are shown in Fig. 5B. The results obtained with anti-VTG2 antibody are shown in Fig. S3.

## DISCUSSION

**Fig. 6.**
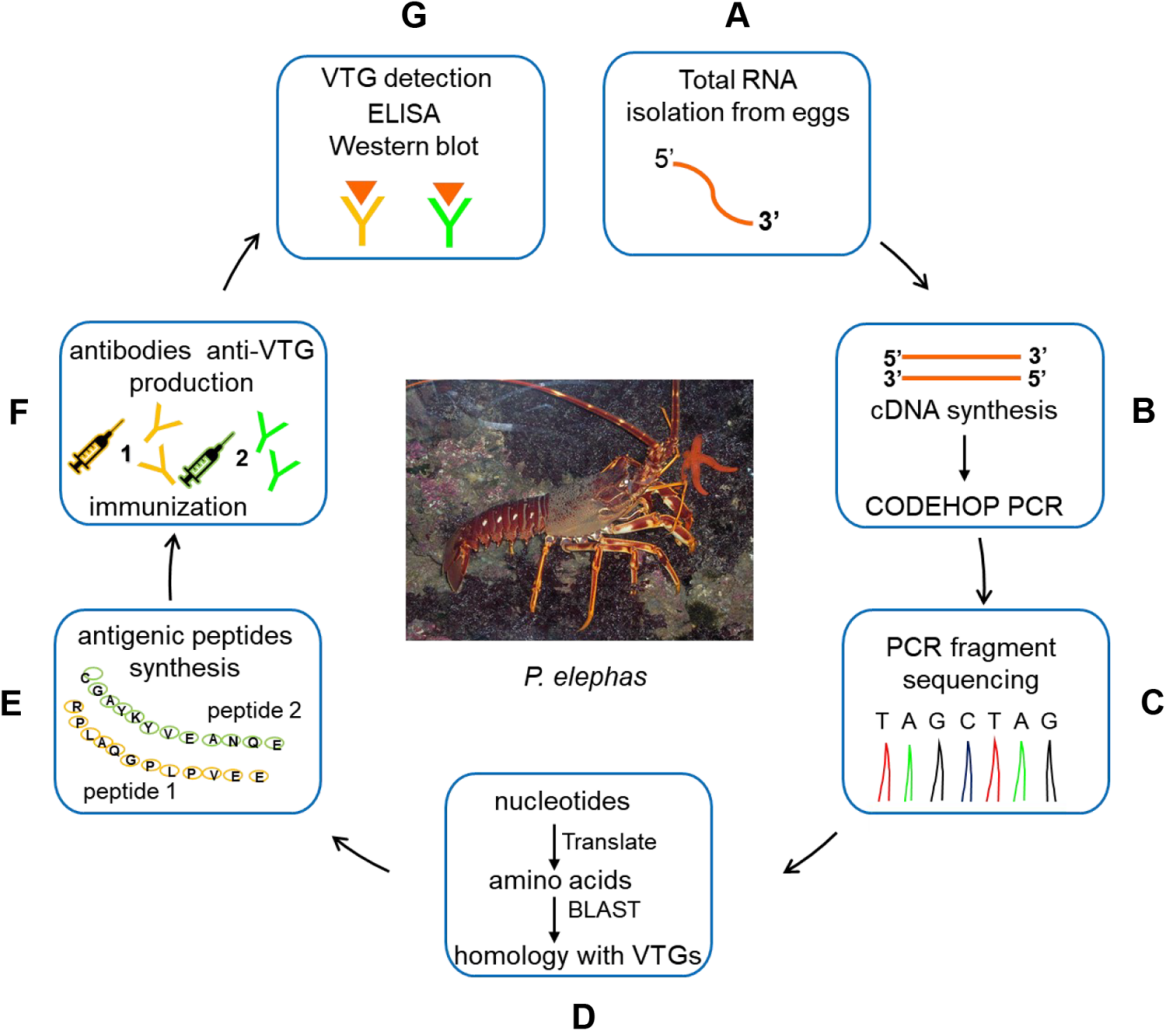
**An overview of steps involved in the development of *P. elephas* anti-VTG antibodies.** (A) Total RNA was extracted from *P. elephas* eggs. (B) To obtain cDNAs, *P. elephas* RNAs were reverse transcribed; to detect the unknown nucleotide sequences of the Vtg gene, CODEHOP PCR was used, starting from aligning the multiple sequences of VTG proteins. (C) The PCR products obtained were used for the preparation of the sequencing samples. (D) The nucleotide sequence acquired experimentally was translated into the amino acid sequence and aligned with protein sequences deposited in NCBI databases. (E) The potential presence of two epitopes in the virtual VTG peptide allowed the biotechnological synthesis of two peptides (peptide 1 and peptide 2), which were used to create two different antibodies for ELISA and western blot experiments (F) to detect the VTG in the females of *P. elephas* in the second stage of egg maturation.

In this work, a study aimed at the creation of anti-VTG antibodies of *P. elephas* was carried out for the first time, starting with the partial isolation of the mRNA coding for VTG. This strategy allowed us to define a part of the primary structure of the protein (GenBank accession number: KX792013.1) and to perform the biotechnological synthesis of two peptides that, due to their high potential, were used to generate two different antibodies. An overview of the steps involved in the development of *P. elephas* anti-VTG antibodies is shown in Fig. 6. Via this molecular pathway, we bypassed the protein purification procedure from tissue or other biological samples in which the protein was expressed, a process that is usually used to generate antibodies (Denslow et al., 1999; Tsukimura et al., 2002; Kang et al., 2008). By means of immunoblotting experiments, we established that both antibodies can recognize three proteins of about 70, 80 and 120 kDa, which, as described in the literature, could originate from the processing of the VTG precursor at the level of two supposed consensus sequences, RXXR or RXK/RK/R, of the subtilisin endopeptidase (Tiu et al., 2009). The immunoreactive band profile of *P. elephas* observed in the western blot experiments is similar to the profile described for mature females of the *H. americanus* lobster, in whose ovaries the presence of three major proteins (of 80, 90 and 105 kDa) that are immunoreactive to a specific anti-HaVn antibody has been identified (Tiu et al., 2009). The authors hypothesized that these proteins probably originated from a precursor that underwent proteolytic cuts at the potential cleavage sites of the subtilisin endopeptidase. The information regarding the primary structure of the *P. elephas* VTG is limited to the 63-amino-acid sequence reconstructed *in silico*. Therefore, it is difficult to hypothesize how many cleavage sites for subtilisin are present in the protein and, consequently, whether the bands we observed could be the result of the complete or incomplete processing that the precursor underwent. The results of the western blot showed that the three proteins detected by anti-VTG1 and anti-VTG2 antibodies could derive from a precursor that underwent two proteolytic cuts. The treatment of the egg homogenate samples with subtilisin did not change the immunoreactive band profile, suggesting that no additional cleavage sites were likely to be present in any of the detected proteins. The western blot and ELISA experiments demonstrated that both antibodies can identify products resulting from VTG processing with the same efficiency.

### Conclusion

This is the first work in which *P. elephas* anti-VTG antibodies were used. For the first time, we examined the content of this protein present in eggs. As reported by Denslow et al. (1999), the quantization of VTG through ELISA represents a powerful tool that can be further utilized in the ecological and marine biological studies of *P. elephas*. In fact, the tool makes it possible to compare the levels observed in individuals of *P. elephas* taken from different repopulation areas, both in protected areas (internal) and in areas dedicated to commercial fishing (external), to identify the possible role of the protein in the reproduction, growth and increase in the number of individuals in the population itself. Moreover, the tool can monitor the concentrations of this protein in individuals grown in both restocking and free areas to ascertain whether any increase in protein levels could be related to the presence of estrogen-like substances in the water. In fact, the use of VTG as a biomarker can indicate not only the presence of a pollutant in the water, but also the effect that it can have on physiological alterations related to the reproductive sphere. The VTG values obtained in the various samples in this study did not highlight any significant differences between the expression levels of mature females living inside and outside protected marine areas. The values found allowed us to establish, for the first time, a concentration range for VTG considering the eggs of females in ovarian stage 2, which is probably correlated with sexual maturity within the same stage of development. The analyses carried out on male individuals did not provide detectable results. A future goal is to analyze a greater number of individuals to constantly monitor any fluctuations in the concentration of this important protein in mature females and males and to determine whether environmental conditions determine VTG expression.

## MATERIALS AND METHODS

### Animals

The Mediterranean *P. elephas* egg clutches samples that were provided by the Marine Biology section of the Department of Life and Environmental Sciences of the University of Cagliari. The animals were captured between October 2018 and March 2019, corresponding to the extruding period of the species (Goñi et al., 2003), inside and outside of outside of two FPAs located on the western coast of Sardinia (Italy) in the middle of the Mediterranean Sea (Fig. 1A). Specifically, the FPAs considered were Su Pallosu located on the central western coast of Sardinia (latitude 40° 3′ 1.1″ N, longitude 8° 24′ 7.9″ E) and Buggerru on its southwestern coast (latitude 39 23′ 48″ N, longitude 8° 24′ 09″ E) (Fig. 1A,B). The concentration of VTG was estimated from the egg clutches of 30 ovigerous females at the same stage of intermediate development (stage 2) as follows: 16 ovigerous females were caught inside of FPAs (60.9–91.9 mm carapace length) and 14 outside of FPAs (68.7–100.3 mm carapace length), all at the same developmental stage (Fig. 1C). The size of external eggs on the pleopods (Fig. 1C) and CL was used as an indicator of maturity (Kang et al., 2008). The developmental stage of the eggs of *P. elephas* (Follesa et al., 2007) was estimated using a binomial generalized linear model (R Core Team, 2017, https://www.R-project.org/) with a logistic link established through evaluating the von Bertalanffy growth parameters calculated for females. The relationship between the egg size at each developmental stage and the female lobster size was evaluated by regression analysis, and significant differences were calculated by *t*-tests (Zar, 1999). VTG levels were also determined in the hepatopancreas and hemolymph of six male *P. elephas*, of which three were captured inside and three were captured outside of the marine protected areas. Once collected, the biological samples were stored at −20°.

### Isolation of total RNA from ovarian tissue and RT-PCR

Total RNA was extracted from 50 mg of *P. elephas* eggs using TRI Reagent (Sigma-Aldrich, St. Louis, MO, USA) following the manufacturer's suggested protocol. The quality of purified RNA was verified by gel electrophoresis using a 1% denaturing agarose gel stained with SYBR Green II (Sigma-Aldrich), and the concentrations were measured using a NanoDrop 2000c UV-VIS Spectrophotometer (Thermo Fisher Scientific, Waltham, MA, USA) at 260 nm. To obtain cDNAs, *P. elephas* RNAs were reverse transcribed with an oligo dT primer using an enhanced avian myeloblastosis virus reverse transcriptase enzyme (Sigma-Aldrich) following the manufacturer's instructions.

### *Palinurus elephas* Vtg cDNA amplification by PCR with hybrid primers

For detecting the unknown nucleotide sequences of the Vtg gene, a degenerate hybrid oligonucleotide primer (CODEHOP) strategy (Rose et al., 2003) was used, starting from aligning the multiple sequences of VTG proteins. In particular, nine different crustacean sources were chosen from the GenBank SwissProt database, which were aligned using Clustal Omega (http://www.ebi.ac.uk/clustalw) (Fig. S1) and then cut into blocks using Block Marker software (Madeira et al., 2019). The primers were designed using the default parameters of the j-CODEHOP server (https://4virology.net/virology-ca-tools/j-codehop/). Amplification primers used for *P. elephas* Vtg cDNA were chosen from a group of primer candidates provided by the j-CODEHOP program (Table S1).

Each primer presents the consensus clamp given in the upper case, whereas the degenerate core is in the lower case: y=[C,T]; *r*=[A,G], and *n*=[A,G,C,T]. PCR was performed in a solution containing 1.5 mM MgCl_2_, 100 mM Tris-HCl, pH 8.3, 50 mM KCl, 200 mM dNTP mix, 1 mM sense primer, 1 mM antisense primer, 1 μg of *P. elephas* cDNA, and one to three units of Jump Start AccuTaq LA DNA polymerase mix (Sigma-Aldrich). Thermal cycles of amplification were carried out in a Personal Eppendorf Mastercycler (Eppendorf, Hamburg, Germany) using slightly different programs. The PCR experiments, conducted using all the pairs of sense and antisense oligonucleotides chosen by j-CODEHOP software, allowed us to obtain a single reaction product with the F3-R2 primer pair (Fig. S1), with dimensions of approximately 300 bp, which was compatible with those of a possible expected product. The PCR products detected on 6% polyacrylamide, or 2% agarose gels were purified with a Charge Switch PCR Clean-Up kit (Invitrogen, Carlsbad, CA, USA) and then sent to BMR genomics (Padova, Italy) for sequencing. Translation of nucleotide sequences was performed using OMIGA or ExPASy translate routine software (http://ca.expasy.org/) (Fig. 2A). Sequences were aligned with Clustal Omega (https://www.ebi.ac.uk/Tools/msa/clustalo/), and similarities were analyzed with the advanced BLAST algorithm available at the National Center for Biotechnology Information website (http://www.ncbi.nlm.nih.gov/) and with the FASTA algorithm v.3.0 from the European Bioinformatics Institute website (http://www.ebi.ac.uk/fasta33/index.htlm).

### Peptide synthesis

The *P. elephas* VTG amino acid sequence deduced in silico (Fig. 2A) was used to create two synthetic peptides (Fig. 2B) (Genscript USA Inc.) to be employed for the production of specific anti-VTG antibodies (Twin Helix, Rho, Italy). The epitopes were predicted by the GenScript Optimum AntigenTM design tool (antigenic peptide 1 AA9-AA23 and antigenic peptide 2 AA50-AA63). Comprehensive analysis was performed on multiple aspects, including antigenicity, hydrophilicity, hydrophobicity, the probability of antibody accessibility (exposure on the protein surface) and the uniqueness of the protein sequence (Kyte and Doolittle, 1982). The useful information related to the synthetic peptides for the synthesis of antibodies is shown in Fig. 2B.

### Total protein quantification

The amount of total protein in *P. elephas* egg lysate was determined via protein quantification using a Pierce™ BCA Protein Assay Kit (Thermo Fisher Scientific, Waltham, MA, USA) and bovine serum albumin (BSA) as a standard. The eggs were sonicated in RIPA buffer (50 mM Tris-HCl, pH 8.0, 150 mM sodium chloride, 1.0% Igepal CA-630 (NP-40), 0.5% sodium deoxycholate, 0.1% sodium dodecyl sulfate) supplemented with a protease inhibitor (Bio-Rad Laboratories, Inc.) in the proportion of 25 mg of eggs per 100 µl of buffer. After homogenization, the tubes were centrifuged at 14,000× ***g*** for 20 min to obtain the supernatant. Standard curve was created by serial dilution of BSA in RIPA buffer. The assay for each sample was carried out according to the manufacturer's instructions. Triplicates were run on each sample and standard. The absorbance was subsequently measured at 562 nm using a Bio-Rad 680 microplate reader. The protein concentration obtained was of about 1.2±0.05 mg/ml for each sample.

### *Palinurus elephas* VTG detection by ELISA

For the detection of VTG, we performed indirect ELISA using the commercial Prepro Tech TMB ELISA Buffer Kit (DBA, Italy) following the manufacturer's suggested protocol. For the standard curve, the antigenic peptides diluted in 2% blocking medium in phosphate buffered saline buffer (PBS, pH 7.4) were added to the wells of the plate at scalar concentrations in the range of 0.97–250 ng per well and then incubated for one hour at 37°C. After three consecutive washes in PBS, the antibodies diluted in 2% blocking medium in PBS at a concentration of 0.1 µg/well were incubated on the plates overnight at 4°C. After the incubation period, the plates were washed five times, and secondary antibodies made of anti-mouse IgG conjugated with HRP diluted in PBS (1:20,000) were added to the wells of the plate for 2 h at 37°C. Finally, the plates were washed six times and then incubated with the tetramethylbenzidine (TMB) substrate HRP. The reaction was carried out in phosphate-citrate buffer (pH 5.5) in the dark for 15 min at room temperature and subsequently blocked with 10% sulfuric acid. The plates were read at a wavelength of 460 nm using the VICTOR 3 V 1420 Multi-Label Microplate Reader (Perkin Elmer, USA). All reactions were carried out in triplicate using both antigenic peptides in separate experiments. The blank samples were composed of buffer or water with no protein sample included. The standard curve R^2^ values obtained were the same for antigenic peptide 1 (*R*^2^=0.9757) and peptide 2 (*R*^2^=0.97). When the homogenate of *P. elephas* eggs was used as an antigen, the ELISA plates were coated with 25 mg of biological sample per well. The eggs were sonicated in RIPA buffer with a protease inhibitor (Bio-Rad Laboratories, Inc.) in the proportion of 25 mg of eggs per 100 µl of buffer (about 1.2 mg/ml of total protein). After homogenization, the tubes were centrifuged at 14,000× ***g*** for 20 min, and the supernatant obtained was used following the steps previously described for the peptides used to construct the standard curve. To validate the ELISA, we determined the intra-assay coefficient of variation (CV %) value, which was less than 15%. The CV was calculated by dividing the standard deviation (σ) of a set of measurements by the mean (µ) of the set (CV %=σ/µ×100). VTG concentration obtained was within the range of 120–180 ng/ml and 200–260 ng/ml (Fig. 4A,B).

### SDS–PAGE

We used a Mini-PROTEAN Electrophoresis System (Bio-Rad Laboratories, Inc.) with 10% Mini-PROTEAN TGX Precast Gel to analyze the anti-VTG antibody samples. SDS–PAGE was performed using a prestained PerfectTM Color Protein Ladder (EURx Ltd. 80–297 Gdańsk, Poland) as the protein marker. A quantity of 5 µl of each antibody solution (0.4 μg/µl) was mixed with 2x Laemmli sample buffer (Bio-Rad Laboratories, Inc.) under non-reducing or reducing conditions in the presence of β-mercaptoethanol (Bio-Rad Laboratories, Inc.) and heated at 95°C for 5 min before gel loading. The antibody bands were detected using a Coomassie Brilliant Blue R-25 Staining Solution (Bio-Rad Laboratories, Inc.). For the SDS–PAGE of the *P. elephas* samples, we pooled an aliquot of egg homogenates considering the two concentration ranges of VTG determined by the ELISA method. Specifically, we pooled homogenate samples with a VTG concentration within the range of 120–180 ng/ml and 200–260 ng/ml. Regarding sample preparation, 5 µl of homogenate of *P. elephas* eggs (25 µg) were diluted with 2x Laemmli sample buffer (Bio-Rad Laboratories, Inc.) under reducing conditions and heated at 95°C for 5 min before gel loading. For the western blot analyses, the homogenates for gel loading were prepared as previously described. Moreover, homogenates for gel loading were prepared after treatment with subtilisin A protease (Sigma-Aldrich). Considering the enzymatic activity of subtilisin reported in the product information, the digestion 1000:1 (protein:subtilisin ratio) was performed at 37°C in 10 mM Trizma buffer (pH 8.0) overnight.

### Western blot analysis

The proteins from SDS–PAGE were transferred onto 0.2 µm nitrocellulose membranes (Bio-Rad Laboratories, Inc.) previously immersed in the transfer solution prepared from Bio-Rad 5x transfer buffer using the Trans-Blot Turbo Transfer System (Bio-Rad Laboratories, Inc.). After the transfer of the proteins, the membranes were rinsed with TBST (100 mM Tris-HCl, 150 mM NaCl, 0.05% [v/v] Tween 20, pH 7.5) and then in the blocking buffer (TBST containing 5% non-fat dried milk) for 45 min at room temperature. The membranes were subsequently incubated with anti-VTG1 or anti-VTG2 antibodies (dilution 1:500) for 1 h. After three washes in TBST, the membranes were incubated for 1 h at room temperature with the secondary antibody anti-rabbit IgG peroxidase conjugate (dilution 1:20,000). Finally, the blots were washed with TBST and developed using enhanced TMB reagents (Sigma-Aldrich) following the manufacturer's instructions.

## Supplementary Material

Supplementary information
