# Supplementary material for: Antibodies targeting the European lobster (Palinurus elephas) vitellogenin developed by mRNA isolation and in-silico-designed antigenic peptides
Source: Biol Open. 2022 May 13;11(5):bio059019. doi: 10.1242/bio.059019 (PMC9116138; doi:10.1242/bio.059019)
Supplement: Supplementary information [file biolopen-11-059019-s1.pdf]

Fig. S1.

Accession numbers

Q1L7X1	***P. trituberculatus***
Q5XXZ5	***C. feriata***
S4SBK7	***E. carini cauda***
A3RKP7	***H. americanus***
Q7Z1G4	***M. ensis***
Q9NDS1	***P. japonicus***
Q2I691	***P. monodon***
A1XFT3	***F. chinensis***
Q6RG02	***P. merguiensis***

CLUSTAL Omega Alignment

```
                                                   F1 →
tr|Q1L7X1|Q1L7X1_PORTR       MTTHTVLLLLALTAAAVAAPYGGTTQLCSTECPLAA-AKLSFIPGKTYSYTYSGKSIVQL
tr|Q5XXZ5|Q5XXZ5_CHAFE       MTTHTV-LLLALAAAAAAAPYGSTIQLCSTECPVAA-AKLAFTPGKTYSYTYTGKSQVQL
tr|S4SBK7|S4SBK7_EXOCA       -MTYSALFVFAILAAASAAPWPSTTNLCSRECPVAGSPKLFYAPEKTYVYSYTGKSRINL
tr|A3RKP7|A3RKP7_HOMAM       MTTSTALLVPPLVVGAGASPWGGNTPRCSTECPVAGSPKLFYQPEHTYTYTYSGKSHIHL
tr|Q7Z1G4|Q7Z1G4_METEN       MNSSSLVLVLALVAGGLAAPREDEAPRCSTECPVTGSPKLAYEPGKTYTYAYSGKSKVQL
tr|Q9NDS1|Q9NDS1_PENJP       MTTSSLLFVLALVAGGLAAPWGADLPRCSTECPISGSPKLAYQPEKTYTYQYSGKSRVQL
tr|Q2I691|Q2I691_PENMO       MTTSTLLFILAFVTGGLAAPWGADLPRCSTECPITGSPKLAYQPDKTYAPPYSGKSRVHL
tr|A1XFT3|A1XFT3_FENCH       MTTSNLLFVLAFVAGGLAAPWGADLPRCSTECPITGSPKLAYQPDKTYAYAYSGKSTVQL
sp|Q6RG02|VIT_FENME          MTTSTLLFVLAFVAGGLAAPWGADVPRCSTECPVTGSPKLAYQPDKTYAYAYSGKSTVQL
                               :     :    ..   :*       ** ***::.  ** : * :**   *:*** :.*

tr|Q1L7X1|Q1L7X1_PORTR       KGVDGGLVETQWEKQMLLTVLGPCDVAISFKGTKVDGKTG-LPGSDKLERYPLVVAMTDG
tr|Q5XXZ5|Q5XXZ5_CHAFE       KGVDGGVVDTRWEEQVLLTVLGPCDLAISFKGSKVDGKTG-LSGSDKLERYPLVVAMTDG
tr|S4SBK7|S4SBK7_EXOCA       KDVEGANTDIEWRSQVELSWLSPCDMAITMKNPSMGGGSG-SSEARFLERYPLVVSIIDG
tr|A3RKP7|A3RKP7_HOMAM       KGVEGGVTGTAWSSQVELSWITPCDVVIFIKNSKVDGAQVSQPAAKFLEKYPMVVALTDG
tr|Q7Z1G4|Q7Z1G4_METEN       KGVQDGDSDIEWAAGIDLTWITPCDVAISFTNIKMDGAPGKPDAARTLQRFPMVVAVAEG
tr|Q9NDS1|Q9NDS1_PENJP       KGVDDGVSETEWAARVDLTWISPCDVAISFNNMKMDGARG-PIAARTLERHPLVVAVVDG
tr|Q2I691|Q2I691_PENMO       KGVDNGDSEIEWTAGVDLTWISPCDMAISFRNTKMDGARG-PIAASTLERHPLVVAVVDG
tr|A1XFT3|A1XFT3_FENCH       KGVDNGDTATEWTAGVDLTWISPCDMAISFRNMKMDGARG-PIAARTLERYPLVVAVVDG
sp|Q6RG02|VIT_FENME          KGVDNGDTETEWTAGVDLTWISPCDMAISFRNTKMDGARG-PTAARTLERYPLVVAVVDG
                             * *: .     *    : *: : ***:.* :    .: *       :  *::.*:**:: :*
                                                                  F2 →
tr|Q1L7X1|Q1L7X1_PORTR       RVQRVCSHPDDDTWAINMKKGVVSALQISLPSLSISNSGLNFTETDVLGTCPTYYEVQGE
tr|Q5XXZ5|Q5XXZ5_CHAFE       RVQRVCSHPDDDTWAINMKKGVVSALQISLPSLSISNSGLNFTETDVLGTCPTYYEVQAE
tr|S4SBK7|S4SBK7_EXOCA       RVMESCAHPDDDVWSVNMKKGIASAFQNTLPSNSSINTGLNFTETDIIGNCSTMYEVKNE
tr|A3RKP7|A3RKP7_HOMAM       RVQHVCTHPEDDVWSINIKKGIASAFQNSLPSNSTINSGQNITETDVVGKCPTHYEVERE
tr|Q7Z1G4|Q7Z1G4_METEN       RVQHVCAHPEDQPWAINLKKGVASALQNSIPSLSPVASGITVTETDVIGKCPPNTKVKTE
tr|Q9NDS1|Q9NDS1_PENJP       RVQHVCAHPDDEPWAINLKKGVASAFQNSIPSLSTVSSGMTVTETDVVGKCPTTYQIETE
tr|Q2I691|Q2I691_PENMO       RVQHVCAHPEDEPWAINMKKGVASAFQNSIPSLSTVSSGITVTETDVVGKCPTKYEIKTE
tr|A1XFT3|A1XFT3_FENCH       RVQHVCAHPEDEPWAINLKKGVASAFQNSIPSLSAVSSGITVTETDVVGKCPTKYEIETE
sp|Q6RG02|VIT_FENME          RVQHVCAHPEDEAWAINLKKGVASAFQNSIPSLSAVSSGITVTETDVVGKCPTKYEIETE

                           **   * **:*: *::*:***:.**:* ::** *   :* ..****::*.*    ::: *
```

```
                                      F3 →                      ← R1
tr|Q1L7X1|Q1L7X1_PORTR           GARVLVKKEKNHRLCKEHYPTPDEINLPYLKGPLPIQESRSICRQEIDSGIISSVVCEDK
tr|Q5XXZ5|Q5XXZ5_CHAFE           GAKVLVKKEKNHRLCQEHYPTPDEIDLPHLKGPLPIQESQSICRQEIDSGIISSVVCEDK
tr|S4SBK7|S4SBK7_EXOCA           GEKVVVMKMKNHRFCQDHFISRAESTKAWLKAPLPMEESFSECKQEITKGVYTSITCKDK
tr|A3RKP7|A3RKP7_HOMAM           GHKVIVKKEKNHRLCKKRYPTPAETQMPWLKGPLPLEESECRCKQEITNGIYSSIICHDK
tr|Q7Z1G4|Q7Z1G4_METEN           GRELIVTKEKEHRHCTEHFPTPNEVPAPWMKAPLPLEESGPECKQVIENGIYTAITCEDK
tr|Q9NDS1|Q9NDS1_PENJP           GEKVIVVKEKNHRHCQQRYPTPAETPAPWLKAPLPIEESKSECKQEITNGIYTSIMCHDK
tr|Q2I691|Q2I691_PENMO           GEKVIVVKEKNHRHCQERYPTPAETPAPWLKAPLPIEESKSQCRQEIANGIYTAITCQDK
tr|A1XFT3|A1XFT3_FENCH           GEKVIVVKEKNHRHCQERYPTPAETPAPWLKAPLPIEESKSQCRQEIANGIYTAITCQDK
sp|Q6RG02|VIT_FENME              GEKVIVVKEKNHRHCQERYPTPAALPAPWLKAPLPIEESKSQCRQEIANGIYTAITCQDK
                                 * .::* * *:** * .:: .        :*.***::**   *:* * .*: ::: *.**
                                        F4 →
tr|Q1L7X1|Q1L7X1_PORTR           KVIRPSYGIYKYVEAKQESTLKLTSSDVSAPDTISRIGQDELVPRSLRYDYEPAKKDPTL
tr|Q5XXZ5|Q5XXZ5_CHAFE           KVIRPSYGMYKYVEAKQESTLRLTSSDVSAPDTISRIGQDELVPRSLRYDYETAKKDPSL
tr|S4SBK7|S4SBK7_EXOCA           NIIRPAYGSYKYIEAVQESTLRFESETDN-VPPAVSQLPSRFIRKTLRYDQHTMKKDPSM
tr|A3RKP7|A3RKP7_HOMAM           KVVRPSYGAYKYVEAKQESTLRYVSLSSQ-Q--PPAIPQGSLVRKSLRYSYHTLNKDPSM
tr|Q7Z1G4|Q7Z1G4_METEN           HIVRPAFGLYKYVEANQESTLRFISESSD-TSAISGITRGELEIESLLFNR-ETTKDPEL
tr|Q9NDS1|Q9NDS1_PENJP           NIVRPAIGIYKYVEANQESTLHFISETTD-TSAITAIPRGEMHIESLLYNH-ETMKDPEL
tr|Q2I691|Q2I691_PENMO           NIVRPAIGIYKYVEASQDSTLRFISESSD-TSAISGIHSGEVHIESLLYNH-ETMKDPQL
tr|A1XFT3|A1XFT3_FENCH           NIVRPAIGIYKYVEASQDSTLRFISESAD-TSAISGIPSGELHIESLLYNH-ETMKDPQL
sp|Q6RG02|VIT_FENME              NIVRPAIGIYKYVEASQYSTLRFISESSD-TSAISGIPSGELNIESLLYNH-ETMKDPQL
                                 .:::*: * ***:** * *.*:  *   .            .  .:* :.     *** :

tr|Q1L7X1|Q1L7X1_PORTR           VPELEQTLRYLCEITRDGVEADTAAQLEKAVNLMRRIPEQSFNDIYTKVRNKQICPQHTK
tr|Q5XXZ5|Q5XXZ5_CHAFE           VPELEQTLTYLCEITKDGVEADVAAHLSKAVHLMRRIPEQGFNEIYTKVHNKQICPQHSR
tr|S4SBK7|S4SBK7_EXOCA           VVKLDAMLKEVCQKMKHGVQEDTASYFANALQYMRRVPEEAIPQTLEKIRGGQICEQRQK
tr|A3RKP7|A3RKP7_HOMAM           VAELDQTMTQICEKTKDVVERDAAALVAKAVQLLRRVPEEAVKQTLDKIRAGRYCQDHSK
tr|Q7Z1G4|Q7Z1G4_METEN           APEIDELMKEICDKTENTVEADAAALVDKALHMLRSVPAEVVEEVAEKVRGGRYCGHSER
tr|Q9NDS1|Q9NDS1_PENJP           APELDQLMKEICEKTKDTVEAEAAALVAKALHLLRRVPETVVVEIAQKVRQGHYCSDSAK
tr|Q2I691|Q2I691_PENMO           APELDELMKEICAKTKDTVEAEAAALVAKALHVLRRVPETIVVETAQKVRQGHYCSDSAR
tr|A1XFT3|A1XFT3_FENCH           APELDELMKEICDKTKDTVEAEAGALVAKALHVLRRVPETVVVETAQKVRQGHYCSDSAR
sp|Q6RG02|VIT_FENME              APELDELMKEICDKTKDTVEAEAGALVAKALHVLRRIPDTVVVETAQKVRQGHYCSDSAR
                                 . :::  :  :*   .. *. ...: . :*:. :* :*   . :   *::  : * .  :
```

```
                                                    ← R2
tr|Q1L7X1|Q1L7X1_PORTR      LESLFMDAIAFVHEPESVPVMVKELVEGRTTGTLAALYSTAFYLVPRPDVKAIQALEPLF
tr|Q5XXZ5|Q5XXZ5_CHAFE      LESMYKDAIAFVHEPESVPVMVKELVEGRATGTLAALYSTGFYLVPRPDVKAIRALEPLF
tr|S4SBK7|S4SBK7_EXOCA      LESMFLDGLAFVYESGAVKVMVQELVSGKATGGRAALYAASMYFMPRPCIHSIEALKPLF
tr|A3RKP7|A3RKP7_HOMAM      LESLFLDAVSFIHESGAVKVMVDELVMARATVSRAALYTAAFYLQPRPCIHAMMALKPMF
tr|Q7Z1G4|Q7Z1G4_METEN      LESIFFDAVAFVHESGAVKVMVEEIENGRATGGRLALYTAALYFTPRPNIKAVEALTPLF
tr|Q9NDS1|Q9NDS1_PENJP      LESIFLDAVAFLHESGAVKVMVQEILNGRATGGRLALYTAALYLTPRPNIEAVKALTPLF
tr|Q2I691|Q2I691_PENMO      LESIFLDAVAFLHESGAVKVMVHEIENGRATGGRLALYMAAVYLTPRPNIEAVKALTPLF
tr|A1XFT3|A1XFT3_FENCH      LESIFLDAVAFLHESGAVKVMVHEIENGRATGGRLALYTAALYLTPRPNIEAVKALTPLF
sp|Q6RG02|VIT_FENME         LESIFLDAVAFIHESGAVKVMVNEIENGRATGGRLALYTAALYLIPRPNIEAVKALTPLF
                            :**:: *.::*::*  :* ***.*:  .::*    *** :..*: *** :.:: ** *:*

tr|Q1L7X1|Q1L7X1_PORTR      KSSADLSSAKLAAASMVNTYCRHKPHCYNETPVRNLAQALKQKIEEDLSSSS-EDTQKQA
tr|Q5XXZ5|Q5XXZ5_CHAFE      KSNADLSSAKLAAASMVNTYCRHKPHCYEESPVRNLAQALKQKIEEDLSSSSREETQKQA
tr|S4SBK7|S4SBK7_EXOCA      EQYQRFPRTTLAGASMVHTYCRQNPKCQEKAPVRQLAETLSSKVGQVCTPSPNEETRKQA
tr|A3RKP7|A3RKP7_HOMAM      ESSQPLPRATLAAASMVNTYCRHNHHCYEEAPVKSLVEALGNKLQRQCSPSADERTQKAA
tr|Q7Z1G4|Q7Z1G4_METEN      ESARPKPTLMLAAASMVNKYCHLVPNCHQEAPVERIARELANKVERHCTPSAGEEAEEEI
tr|Q9NDS1|Q9NDS1_PENJP      ESPRPMPSLLLAAASMVNHYCRHTPHCHQEAPVERIAEILAAKVEGHCSPSIGVEEKEEA
tr|Q2I691|Q2I691_PENMO      EALKPSPSLLLAAASMVNNYCRHTPACYEEAPVARIAEILATRVQSHCSPSAGAEDKEVA
tr|A1XFT3|A1XFT3_FENCH      ESPRPMPSLLLAAASMVNNYCRHTPACYEKAPVAGIAEILANRVQSHCSPSAGVEDKEVA
sp|Q6RG02|VIT_FENME         ESPRPMPSLLLAAATMVNHYCRHTPACYEKAPVARIAEILANRVQTHCSPSAGVEDNEVA
                            :         **.*:**. **:    * :::**  :.. *  ::    : *     .:
                                F5 →
tr|Q1L7X1|Q1L7X1_PORTR      LSALKSLGNMGVMTPEVAEKVVLYMENENKKVSARSRC-TSLQADRVS---RPVTQKLVH
tr|Q5XXZ5|Q5XXZ5_CHAFE      LSALKSLGNMGVMTPETADKVILYMENENKKVSTRVAAAQAFRLTKCQ---RLVTQKLVQ
tr|S4SBK7|S4SBK7_EXOCA      LVLLKSLGNMGVMNSEMARPIMQCIENSEADQGIRIAATQAFRNVRCTPEIHPAIKQLIN
tr|A3RKP7|A3RKP7_HOMAM      LATLKTLGNMGVMTQEVARFVMTCVDTEGVKINVRVAAAQAFRQVKCE---RHQTEHLVN
tr|Q7Z1G4|Q7Z1G4_METEN      VAALKALGNMGVVTPAVTSAAVTCIEEHEVPTSIRVAAAEVFRQAKCY---RPAVEKLVD
tr|Q9NDS1|Q9NDS1_PENJP      LAIFKALGNMGVVTPAVTRAAAQCIEKEGLETSIRVAAAQAFRQANCD---RPAVQKLVD
tr|Q2I691|Q2I691_PENMO      LAIFKAVGNMGVITPAVTRAAVACIEQEGVETSIRVAAAHVFRQTKCY---RPAVEKLVS
tr|A1XFT3|A1XFT3_FENCH      LAIFKAIGNMGVATPAVTRAAVHCIEEEGLETSIRVAAAQAFRQANCF---RPAVEKLVD
sp|Q6RG02|VIT_FENME         LAIFKTIGNMGVATPAVTRAAVHCIEVEGLETSVRVAAAEAFRQANCF---RPAVEKLVD
                            :  :*::***** .   :      ::        *  .   ::  .     :   ::*:
```

Biology Open • Accepted manuscript

```
                                                                              ← R3
tr|Q1L7X1|Q1L7X1_PORTR              YALRPEENTEVRIAAYLAAVRCANYEDLQEIVTKISY---EENTQVRGFILSHLINLQKS
tr|Q5XXZ5|Q5XXZ5_CHAFE              YALRPGQNTEVRIAAYLAAVRCANYEDLQHIVTKISY---EENTQVRGFILSHLINLQQS
tr|S4SBK7|S4SBK7_EXOCA              VVLDPKKKTEVRIGSYLAAVKCAKYEDLKKITDKIAI---AENTQVRSFILSHLQNVRES
tr|A3RKP7|A3RKP7_HOMAM              IAVHPAKNTEFRIASYLMAMRCVRQKDLQMIVNNTYHEKHSKNTQVRGFILSHLLNLQDT
tr|Q7Z1G4|Q7Z1G4_METEN              IATHPDFETEVRIVSYLAAIKCAEMEDLEKIINKITE---EKNTQVRSFVLGHLINIQES
tr|Q9NDS1|Q9NDS1_PENJP              IATRPTFETEVRIASYLAAIRCAEKEHLEQIIEKISE---EENTQVRGFVLGHLINIQES
tr|Q2I691|Q2I691_PENMO              IAVRPAFGTEVRIASYLAAIRCAEMEDLEEIFEKISV---EENTQVRGFILGHLLNIQES
tr|A1XFT3|A1XFT3_FENCH              IAVRPAFETEVRIASYLAAVRCAEQEHLETIIEKISK---EENTQVRGFVLGHLINIQES
sp|Q6RG02|VIT_FENME                 IAVRPAFETEVRIASYLAAVRCAEQEHLETIIEKISK---EENTQVRGFVLGHLINIQES
                                     .      **.** :** *::*.. :.*. *  :       :*****.*:*.** *::.:

tr|Q1L7X1|Q1L7X1_PORTR              DAPEKQSLRYMMTNIVLPQDFEADIRKYSQNLDLSYFSESLGVGAEVESNLIYAPGSMIP
tr|Q5XXZ5|Q5XXZ5_CHAFE              DAPGKQRLRYLLTNILLPQDFETDIRKYSQNLDLSYFSPSA---AGLESNMIYAPGSMLP
tr|S4SBK7|S4SBK7_EXOCA              TASFKGNLKNMLETIVLPSNFTKDWRKISRNVDLSYYAPTFGVGAGMESNLIYAPGSFVP
tr|A3RKP7|A3RKP7_HOMAM              NTPHKDYLRYLLTNTLLPRDYKTDFRKYSRNIDMSYFAPSLGVGAGVESNIIYVPGSFVP
tr|Q7Z1G4|Q7Z1G4_METEN              TCPSKEHLRYLLTDFVIPTDFERDFRRVSRNVMMGYHSAAFEMGADVESNIIYAPGAFVP
tr|Q9NDS1|Q9NDS1_PENJP              TCPTKENLKYLLTNVVIPTDFEKDFRKFSRNVEMSYHAPAFGMGADLESNIIYAPGSFIP
tr|Q2I691|Q2I691_PENMO              TCPTKEHLRYLLTNFVIPIDFERDFRKFSRNIDVAYHAPAFGMGAGLESNIIYAPGSFIP
tr|A1XFT3|A1XFT3_FENCH              TCPAKENLRYLLANVVIPTDFERDFRKFSRNIDMAYHAPAFGMGAGLESNIIYAPGSFVP
sp|Q6RG02|VIT_FENME                 TCPAKENLRYLLANV-IPTDFERDFRKFSRNIDVAYHAPAFGMGAGLESNIIYAPGSFVP
                                        *  *:  :    :* ::  * *: *:*: :.*.: :    * :***:**.**: :*
                                                             F6 →
tr|Q1L7X1|Q1L7X1_PORTR              RSLGVNLTAALDGTGIPMNLGEIGARLEGLEPILAQLLGPASYLKTSSYSKMFNDLVSFI
tr|Q5XXZ5|Q5XXZ5_CHAFE              RSLGVNLTAAPDGTGIPINIADVGARVEGLEPIIAQLLGPASYLKTSSYSKIFNDLVSFI
tr|S4SBK7|S4SBK7_EXOCA              RSVNLNLTGALGA--TPFNIGEIGARFEGIESIIEEMFGPESYLRKTSSRQILRDLSSNI
tr|A3RKP7|A3RKP7_HOMAM              RSVDFNFTAAFEG--ISMNIGEVGARLEGLEPVIEEVFGPEGYLQRTSFSQILTDMTTFV
tr|Q7Z1G4|Q7Z1G4_METEN              RALTMNLKADIDE--THLDIGEIGARFEGVDSIIEEHFGPEGYLRKASFGKILEDITGFA
tr|Q9NDS1|Q9NDS1_PENJP              RAVNLNMKAAVDE--THMDLAEIGARFEGIDSIIEELFGPEGYLRKATFGKIMQDITGFA
tr|Q2I691|Q2I691_PENMO              RAVNLKMKADVDE--MHMDIAEIGARFEGIDSIIEELLGPQGYLHRATFGKIMEDITGFA
tr|A1XFT3|A1XFT3_FENCH              RAVNMKMKADVDE--THMDIAEIGARFEGVDSIIEELLGPQGYLRRATFGKIMEDITGFA
sp|Q6RG02|VIT_FENME                 RAVNLKMKADVDE--THMDIAEIGARFVGIESIIEEPLGPQGYLRTATFGKIMEDITGFA
                                    *:: .::..        :::.::***. *:: :: : :** .*:: :   ::  *:
```

```
tr|Q1L7X1|Q1L7X1_PORTR              QKNWSTIKQELEVAIRERRSVDYAALESIISKLYGPHYGKFQADFFARFLGQEINYASLS
tr|Q5XXZ5|Q5XXZ5_CHAFE              QQNWSTIKQELEVAIRERRTVDDATIESIISKLYGPQYGQFQADFFARFLGQEINYASLS
tr|S4SBK7|S4SBK7_EXOCA              EETFNKINERLQGSFRQRRSIDLSQISHLFDKLYGNRH-MQKADFYARINNQEMAFGSLM
tr|A3RKP7|A3RKP7_HOMAM              EQKGNKILEKLQQDLRQKRSIDLSTLINFLGNLYSDQSRVTRADVFARFMGQEITFASLA
tr|Q7Z1G4|Q7Z1G4_METEN              EEKGYKVMEHLENTLRTRRSIDASTIADFFNKLYGERASDVRAEVFARIMGQEVTYANIA
tr|Q9NDS1|Q9NDS1_PENJP              EEKGLKVMEHIKQTLRTKRSIDSSVISDFFGKLYGEGRSHTHAEVFARIMGHEITYADVA
tr|Q2I691|Q2I691_PENMO              GEKGLKVMEHIKHTLRTRRSIDSSVISDFFGKLYGEGRSHVHADLFARFMGHEITYADVA
tr|A1XFT3|A1XFT3_FENCH              GEKGLKVMEHIKGTLRTRRSIDSSVIADFFSKLYGESRSHTHAELFARFMGHEITYADVA
sp|Q6RG02|VIT_FENME                 GEKGYKVMEQLKHTLRTRRSIDSSVIADFFGKLYGKSRSHTHAELFARFMGHEITYADVA
                                     :.  .: :.::  :* :*::* : :  :: :**.      :*:.:**:  :*: :..

tr|Q1L7X1|Q1L7X1_PORTR              DNLQDINIQHLVEASVRYLKRMLSSLKNMDLDMVKA-AQLGVDYSL-PTIQGTPLKMKLE
tr|Q5XXZ5|Q5XXZ5_CHAFE              EHLQDINMHHLVEASVRCPMQMLSSLKNVNLDKVKA-VQLGVDYSF-PTIQGTPLKITSE
tr|S4SBK7|S4SBK7_EXOCA              GNMKNIKMEELINNMFDSFDDMINRAAENNLDTVRA-AQLYLDYHL-PTMQGLPLKMKLE
tr|A3RKP7|A3RKP7_HOMAM              GDLKHISADEMIESFFSYFDDIPPQMKNLAHQFQHERLKLTLDYHLSPPSRAYHSKLQLN
tr|Q7Z1G4|Q7Z1G4_METEN              ETLKGVTADKIIETFFSLVDDSLEELKGLNLNTSKELLKLHLYYSL-PTIQGLHSRSPWK
tr|Q9NDS1|Q9NDS1_PENJP              ESLKGVTADTLIETFFSFFEESLEQMKGLNLNTART-AQLYMDYSL-PTIQGTPLKLKLA
tr|Q2I691|Q2I691_PENMO              ESLKGVTADTLIETFFSFFENSLEHMKDLNLNTART-AQLSMDYSL-PDIQGTPLRLKLA
tr|A1XFT3|A1XFT3_FENCH              ESLKGVTADTLIETFFSFFENSLEHMKDLNLNTART-AQLSMDYSL-PTIQGTPLRLKLA
sp|Q6RG02|VIT_FENME                 GSLKGVTADTLIETFFSFFENSLEHMKDLNLNTART-AQLSMDYSL-PTIQGTPLRLRLA
                                      :: :. . :::  .               :  :  :: :   : *  :.   :

tr|Q1L7X1|Q1L7X1_PORTR              TVAVAGIKMQTNLNGL--FSGQGSSGSLFKILPSFSVETHGFIGYDAYISKSGLKMNTTV
tr|Q5XXZ5|Q5XXZ5_CHAFE              TMAVAGIKMETNLNGL--FSGQGGSESKLKILPSFSVETHGFIGYDAYISKSGLKMNTTV
tr|S4SBK7|S4SBK7_EXOCA              GTAMVGLKMESRVRGL-----MSGTPGVMKFYPSLSTQIDAFIGYDCHIVRTGIKMRNRI
tr|A3RKP7|A3RKP7_HOMAM              GKAVGGLKMQGNLNLIDILFNWRNGENLFKIIPSLSVQVDGFVGYDSYNSKLGLEMRNTI
tr|Q7Z1G4|Q7Z1G4_METEN              GTAVSGLKMEGNFHIAHILANPGNLDTAIKLYPGLSIHTTGFVGFDAFIAKAGIEMKNTI
tr|Q9NDS1|Q9NDS1_PENJP              GTAVAGLKMEGDFNIAQILSDPGNLQTGIKLFPALSVQATGFVGFECRLTRVGIEMENTI
tr|Q2I691|Q2I691_PENMO              GTAVAGVKMEGNVNIGQILSDLGNSQTGIKFFPGLSVQATGFVGFACGFTRVGIEMQNTI
tr|A1XFT3|A1XFT3_FENCH              GTAVAGLKMEGNINVAQILSDLGNSQTGVKFFPGLSVQATGFVGFECRLTKVGIEMQNTI
sp|Q6RG02|VIT_FENME                 GTAVAGLKMEGNVNIAQILSDLGNSQTGVKFFPGLSVHATGFVGFDWLLARVGIEMQNTI
                                      *: *:**:  ..              .*: *.:* .  .*:*:     : *::*.. :
```

```
                                                                     ← R4
tr|Q1L7X1|Q1L7X1_PORTR        SSNNGVAIKVGGQSSQEVQIEVDLPKKMEIIRVQSETYLKKQTRNQP-EIKILPPSMQDI
tr|Q5XXZ5|Q5XXZ5_CHAFE        SSSNGVAIKVGGQSSQELQIEVDIPDKMEIIRVHSETFLMKHHENTSQTTRSCQPSMQDI
tr|S4SBK7|S4SBK7_EXOCA        STNVGTSINAKYTSGQGFEVTLEIPEKMELLNMRSEIYLMKRVMGQE-ETKINPSSVRST
tr|A3RKP7|A3RKP7_HOMAM        SSSNGVSIMVKPKNANELELHLDVPVKMDLIAVKSQIYMMKQIRGQP-RTEISPPSSEGG
tr|Q7Z1G4|Q7Z1G4_METEN        SSATGAAIKLRTTDNKKIEMELELPEKMELINIKAETYLVKAV-GKN-VRKIAPSSMRDE
tr|Q9NDS1|Q9NDS1_PENJP        SSATGASINIRTTENKKIQMELEIPEKMELLNIKAETYLVKAV-GKK-LTKITPPTVRDV
tr|Q2I691|Q2I691_PENMO        SSATGAAIKIRTTENKKIEMELEIPERMELLNIKAETYLVKAV-GKK-ITKISPSSMRDV
tr|A1XFT3|A1XFT3_FENCH        SSATGAAIKIRTTENKKIEMELDVPEKMELLNIKAETYLVKAV-GKK-MTKISPSSMRDV
sp|Q6RG02|VIT_FENME           SSATGAAIKIRTTENKKIEMELEVPEKMELLNIKAETYLVKAV-GKK-MTEISPSSMRDV
                              ::  *.:*     . : .:: :::* :*:::  ::: :: *         .    : .
```

Fig. S1. Multiple alignment of VTG amino acid sequences from nine different crustacean sources. The sequences were chosen from the GenBank SwissProt database and aligned using Clustal Omega (https://www.ebi.ac.uk/Tools/msa/clustalo/). The figure shows the alignments based upon which the CODEHOP primers have been designed. An asterisk (*) denotes identical residues; a colon (:) denotes a conserved residue substitution; a full stop (.) denotes partial conservation of the residue. The arrows above the amino acid sequences indicate the position of the sense (F →) and antisense (← R) primers chosen from a group of candidate primers obtained from the j-CODEHOP programme. The sequences of the sense and antisense primers, shown inside the boxes, were obtained from the Clustal Omega alignments

Biology Open • Accepted manuscript

Clustal Omega alignment

```
P.elephas       -----------------TL-PTQLRSRPLAQGPLPVEESGSVCLQEIKDGIYWSITCEDE   42
QDE13857.1      GEKVVVTKLKNHRFCQDHYANRAETPKAWMKAPLPMEESYSECKQEITNGIYTSITCKDK   112
ABO09863.1      GHKVIVKKEKNHRLCKKRYPTPAETQMPWLKGPLPLEESECRCKQEITNGIYSSIICHDK   240
                                              :.***:*** . * ***.:*** ** *.*:

P.elephas       NVIRPSYGAYKYVEANQESTT---------------------------------------   63
QDE13857.1      NVIKPAYGSYKYVEAHQESVLRFQSETDQIPPSVSQLPSRFIRKTLRYDQHTLKKDPSMA   172
ABO09863.1      KVVRPSYGAYKYVEAKQESTLRYVSLSSQQPPAIPQ—GSLVRKSLRYSYHTLNKDPSMV    298
                :*::*:**:******:***.


QDE13857.1     Macrobrachium potiuna   (69.39% identity)
ABO09863.1     Homarus americanus      (67.92 % identity)
```

Fig. S2. Alignment of VTG *P. elephas* amino acid sequences with homologous VTG sequences from GenBank. Identity and similarity percentages are shown at the top of each Clustal Omega alignment panel. Numbers on the left indicate the positions of the amino acids in each protein. An asterisk (*) denotes identical residues; a colon (:) denotes a conserved residue substitution; a full stop (.) denotes partial conservation of the residue.

Biology Open • Accepted manuscript

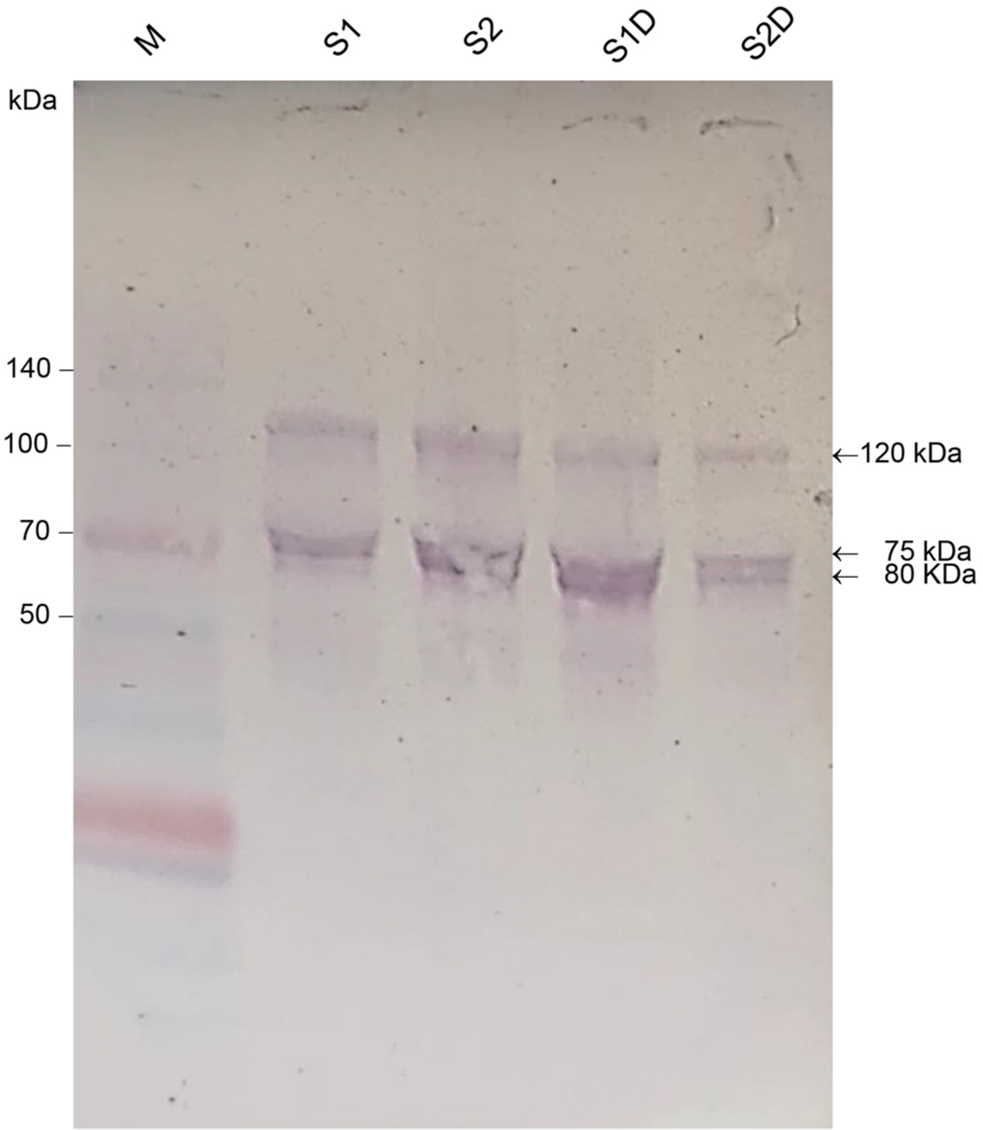

Fig. S3. Results of Western blot analysis using anti-VTG2 antibody. An aliquot of all homogenate samples with concentration of VTG120-180 ng / mL were pooled into one sample (S1). Similarly, an aliquot of all samples with a concentration of VTG 200-260 ng / mL (S2) was pooled. The same S1 and S2 samples were also digested with the subtilisin protease (S1D and S2D samples). Western Blot profiles did not highlight particular differences between the samples treated and untreated with the protease. The different concentrations of VTG detected by ELISA were also not reflected in the number of immunoreactive bands to the anti-VTG2 antibody. The arrows (←) indicate the presence of an immunoreactive band of about 120 kDa and also two very close bands between 80-75 kDa.

Biology Open • Accepted manuscript
